# Adaptation and qualitative evaluation of Ask 3 Questions — a simple and generic intervention to foster patient empowerment

**DOI:** 10.1111/hex.13114

**Published:** 2020-08-01

**Authors:** Anja Lindig, Pola Hahlweg, Wiebke Frerichs, Cheyenne Topf, Martin Reemts, Isabelle Scholl

**Affiliations:** ^1^ Department of Medical Psychology University Medical Center Hamburg‐Eppendorf Hamburg Germany; ^2^ Center of Health Care Research University Medical Center Hamburg‐Eppendorf Hamburg Germany

**Keywords:** patient empowerment, patient participation, qualitative methods, question prompt list, shared decision‐making

## Abstract

**Background:**

Patients are often not actively engaged in medical encounters. Short interventions like Ask 3 Questions (Ask3Q) can increase patient participation in decision‐making. Up to now, Ask3Q was not available in German.

**Objective:**

To translate Ask3Q and evaluate its acceptability and feasibility.

**Methods:**

We translated and adapted several English versions of Ask3Q using a team translation protocol and cognitive interviews. Acceptability and feasibility of the final German Ask3Q version were assessed via focus groups and interviews with patients and healthcare professionals (HCPs). Data were analysed via qualitative content analysis.

**Results:**

Translation and adaptation were successful. Participants of focus groups and interviews perceived Ask3Q as a tool to empower patients to ask more questions. Moreover, it was seen as a guideline for physicians not to forget conveying important information. Several characteristics of patients, HCPs, the clinical setting and the intervention were identified as facilitators and barriers for an effective implementation of Ask3Q.

**Conclusion:**

We provide the German version of Ask3Q. According to participants, implementation of Ask3Q in the German healthcare system is feasible. Future studies should evaluate if positive effects of Ask3Q can be replicated for patient participation and communication behaviour of HCPs in Germany.

**Abbreviations**
AL: Anja Lindig
CT: Cheyenne Topf
IS: Isabelle Scholl
MR: Martin Reemts
PH: Pola Hahlweg
WF: Wiebke Frerichs

**SHORT INFORMATIVE**

- Short question prompt lists like Ask 3 Questions (Ask3Q) can increase patient participation and shared decision‐making.
- We provide the first Ask3Q intervention in German language.
- Ask3Q has the potential to motivate patients to ask more questions in a clinical encounter and can be used as a guideline for physicians to not forget to convey important information.
- According to participants of this study, implementation of Ask3Q in the German healthcare system is feasible if facilitators are considered.
- Future studies should evaluate if effects of Ask3Q can be replicated on patient participation in decision‐making, question‐asking behaviour of patients and communication behaviour of healthcare professionals in Germany.

## 1 BACKGROUND

Adequate communication and information exchange between healthcare professionals (HCPs) and patients are important preconditions for patient participation in decision‐making, also known as shared decision‐making (SDM).^1^, ^2^ If patients are actively involved in decision‐making processes, they know more about their disease and treatment, have more realistic expectations regarding treatment effects, increased trust in their HCP, better risk perception and less decisional conflict.^3^

Most often, there is an asymmetry in patient‐physician interactions.^4^ Many patients experience lower levels of participation in decision‐making than they wish for.^5^ Physicians often dominate the verbal exchange within medical encounters,^6^ rarely invite patients to ask questions and often use closed questions.^7^ On the other hand, patients see a need of adopting the role of a ‘good patient’ and avoid questioning physicians recommendations to not challenge their authority.^8^ In the context of multidisciplinary care, nurses play an important role in integrating the medical, social and lived experiences of patients through dialogue and sharing of knowledge.^9^, ^10^, ^11^ Thereby, they can support patients preferences for participation and can deliver care according to those preferences.^12^, ^13^, ^14^ Despite this potential benefit, an observational study by Hahlweg et al.^11^ showed that nurses were rarely involved in the decision‐making process. There is a need to integrate the expertise of nurses more comprehensively and to enhance nurse‐patient communication to foster SDM in routine practice.^10^, ^15^, ^16^, ^17^

Therefore, we need interventions to both empower patients to actively engage in medical encounters, ask more questions, and provide more information about their values and preferences^18^, ^19^, ^20^ and improve communication skills of HCPs, including physicians and nurses.^21^ Tools like generic question prompt lists (QPLs) consist of only a few core questions and encourage patients to ask them during consultations.^18^ They increase patients’ knowledge and accuracy of risk perception, and reduce the proportion of passive or undecided patients^18^, ^20^, ^22^ without affecting consultation length.^23^, ^24^

The QPL Ask 3 Questions (Ask3Q) consist of three questions regarding treatment options and the decision‐making process.^23^ Slightly different versions of this simple intervention were used in international intervention studies.^25^, ^26^, ^27^, ^28^, ^29^ Ask3Q leads to more SDM by increasing patients’ question‐asking behaviour, physicians’ provision of information about treatment options, their risks and benefits, and integration of preferences in decision‐making.^25^, ^26^, ^27^, ^29^

Up to now, Ask3Q was not available in German. Aim of this study was to translate Ask3Q and evaluate its acceptability and feasibility by patients, physicians and nurses.

## 2 METHODS

### 2.1 Study design

We conducted a two‐phased qualitative study. In the first phase, we developed a German Ask3Q by translation and adaptation of English Ask3Q versions. In the second phase, we assessed acceptability and feasibility of the German Ask3Q by focus groups and individual interviews. To report this study, we used the Consolidated criteria for reporting qualitative studies (COREQ, see Data S1).

### 2.2 Phase 1: Translation and adaptation

#### 2.2.1 Translation

We translated the seven different currently available versions of Ask3Q^23^, ^28^, ^30^, ^31^, ^32^, ^33^, ^34^, ^35^ by following the team translation protocol TRAPD (Translation, Review, Adjudication, Pretesting and Documentation^36^), a method with growing recognition within translation research.^37^, ^38^ One version of the title, four versions of the introduction, two versions of framing sentences, two versions of question 1, four versions of question 2 and five versions of question 3 were available for translation. First, two members of the study team (PH, AL), proficient in German and English, independently translated these versions into German. Second, a third bilingual team member (IS) reviewed the translated versions of PH and AL and either choose one of them or suggested a third version. Finally, PH, AL and IS discussed all translations and suggestions until reaching consensus on a final translation for the Ask 3 Questions intervention to be further tested for comprehensibility.

#### 2.2.2 Adaptation

To assess comprehensibility of all translated Ask3Q versions, we conducted cognitive interviews with a convenience sample of patients with a cancer disease.^39^, ^40^ Cognitive interviewing is a method for pretesting translations in cross‐cultural adaptation studies.^41^, ^42^, ^43^ It helps to evaluate if the content to be tested is understood like its author intends.^42^ Because of the convenience sampling approach, reaching theoretical saturation was not intended and difficult to obtain. Nevertheless, we could observe saturation in feedback and suggestions of our participants. Participants were recruited via outpatient clinics of a Comprehensive Cancer Center in Hamburg, Germany. A member of the study team (AL) personally invited participants to take part in the study. For details about researcher characteristics, the recruitment process and the setting of data collection, see Data S2. We developed an interview guide based on recommendations by Willis et al.^39^, ^41^ Participants were asked for their comprehension of the different versions of the translated title, introduction, framing sentences and the three questions. We used verbal probing techniques like comprehension probes (eg ‘What does the term ‘healthcare’ mean to you?’) and paraphrasing (eg ‘Can you repeat this sentence in your own words?’). We also asked which version of ASK3Q participants would find most useful during clinical encounters and for suggestions for further improvement of the different sentences and questions. We assessed demographic, clinical data, and health literacy (HLS‐EU‐Q16^44^) and calculated descriptive statistics using SPSS (IBM SPSS Statistics, Version 23). Participants were offered a compensation of 25 Euros. Interviews were conducted by AL, audio‐recorded and transcribed verbatim. The study team (AL, PH, IS) discussed the results and selected a final version of the title, the introduction, the framing sentences and the three questions, which were understood best and considered most useful by most of the participants.

The German Ask3Q was designed in postcard and poster formats in collaboration with the University Medical Centers’ graphics department (see Data S3).

### 2.3 Phase 2: Assessment of acceptability and feasibility

#### 2.3.1 Data collection

To assess acceptability and feasibility of the German Ask3Q, we conducted focus groups with patients with a cancer disease, physicians and nurses, using a convenience sampling approach. Nurses and physicians, who were interested in participation but could not take part at the announced dates, were offered an individual interview. Participants were recruited via inpatient and outpatient clinics of a Comprehensive Cancer Center and outpatient oncology practices in Hamburg, Germany. A member of the study team (AL) invited participants to take part in the study either personally, via mail, e‐mail or phone. For details about researcher characteristics, the recruitment process and the setting of data collection see Data S2. Each focus group was moderated by two researchers (AL, IS, PH, WF). Interviews were conducted by AL. Participants were offered a compensation of 25 Euros. All focus groups and interviews were audio‐recorded and transcribed verbatim. We assessed demographic data of all participants, as well as clinical data, the Control Preference Scale (CPS^45^) and health literacy (HLS‐EU‐Q16^44^) of patients.

#### 2.3.2 Focus groups and interview guideline

Focus groups and interviews followed a semi‐structured guideline ^46^, ^47^, ^48^, which was not pilot tested. After a short introduction of the concept of Ask3Q, participants were asked about (1) acceptability (eg for patients: ‘Would you like to use these materials in a clinical consultation?’, for physicians/ nurses: ‘Would you like to use these materials in your daily work?’) and (2) feasibility (eg for all participants: ‘Which conditions must be met in hospitals and practices, to use the material for patient empowerment in a reasonable way?’). Slightly different questions were used for focus groups with patients and HCPs (see Data S2).

#### 2.3.3 Data analysis

We calculated descriptive statistics using SPSS (IBM SPSS Statistics, Version 23). Transcripts were analysed using qualitative content analysis.^46^, ^49^, ^50^, ^51^, ^52^ Two team members (AL/MR) shared the task of primary coding of all transcripts and created a coding scheme with subcodes. Codings and coding scheme were discussed with a second team member (CT/AL) until reaching consensus. The coding scheme was slightly adapted after rereading all transcripts (AL) and discussion within the study team (AL, IS, PH). Analysis was facilitated using MAXQDA 12 (Verbi GmbH).

## 3 RESULTS

### 3.1 Phase 1: Translation and adaptation

#### 3.1.1 Translation

Translators (PH, AL, IS) did not differ much in their translations. Only slight differences in the sentence structure or single words without differences in meaning could be observed. We reached consensus for translation of all Ask3Q versions within the first round of discussion (see Data S4). For cognitive interviews, we combined the different translated versions of the introduction and tested one introduction for the inpatient setting and one introduction for the outpatient setting. We combined the translations of the framing sentences and tested one version. We also combined two versions of question 2 because they only differed in one word (‘harms’ vs. ‘risks’), which was translated to the same German word.

#### 3.1.2 Adaptation

We conducted cognitive interviews with n = 10 patients with a cancer disease. Interviews lasted 39.02 minutes on average. For demographic and clinical data of the participants, see Table 1.

**Table 1:** Demographic and clinical data of participating patients of cognitive interviews (n = 10) and focus groups (n = 24) (SD = standard deviation)

	Phase 1: Patients of cognitive interviews (n = 10)	Phase 2: Patients of focus groups (n = 24)
	n (%)	n (%)
Age (in years)		
Mean (SD)	49.0 (11.3)	60.7 (11.8)
Range	31‐64	29‐74
Gender		
Female	5 (50.0)	13 (54.2)
Male	5 (50.0)	11 (45.8)
Mother tongue		
German	9 (90.0)	23 (95.8)
Other	1 (10.0)	1 (4.2)
Education^a^		
Low	2 (20.0)	3 (12.5)
Medium	3 (30.0)	6 (25.0)
High	5 (50.0)	15 (62.5)
Current Profession^b^		
Employed	5 (50.0)	11 (45.8)
Retired	3 (30.0)	11 (45.8)
Homemaker	1 (10.0)	1 (4.2)
Student/Trainee	1 (10.0)	0 (0.0)
Sick leave	0 (0.0)	2 (8.3)
Cancer entity^b^		
Leukaemia/Lymphoma/Myeloma	3 (30.0)	7 (29.2)
Prostate cancer	0 (0.0)	5 (20.8)
Breast cancer	2 (20.0)	4 (16.7)
Bladder cancer	0 (0.0)	2 (8.3)
GIST^c^	0 (0.0)	2 (8.3)
Testicular cancer	0 (0.0)	2 (8.3)
Ovarian cancer	2 (20.0)	1 (4.2)
Colorectal cancer	1 (10.0)	0 (0.0)
Sarcoma	1 (10.0)	1 (4.2)
Squamous cell carcinoma	1 (10.0)	0 (0.0)
Years of disease		
Mean (SD)	4.8 (3.55)	4.99 (4.8)
Range in years	1‐11	0.42‐19.25
Health literacy^d^		
Inadequate	1 (10.0)	7 (29.2)
Moderate	4 (40.0)	10 (41.7)
Adequate	5 (50.0)	7 (29.2)

^a^ low = <10 school years, medium = 10‐13 school years, high = >13 school years.

^b^ more than one answer possible.

^c^ gastrointestinal stromal tumour.

^d^ health literacy was assessed by HLS‐EU‐Q16, according to the sum score, participants can be divided into three groups: 0 to 8 = inadequate health literacy, 9 to 12 = moderate health literacy, 13 to 16 = adequate health literacy

In cognitive interviews, we tested one version of the title, two versions of the introduction, one version of the framing sentence and question 1, three versions for question 2 and five versions for question 3 (see Data S4). According to suggestions of participants, we added the word ‘important’ (German: ‘wichtige’) to the title of the German Ask3Q. Some participants were not sure about the correct meaning of the phrase ‘about your healthcare’ (German: ‘über Ihre Gesundheitsversorgung’); therefore, we changed that phrase in the final version of the introduction to ‘about your further treatment’ (German: 'über Ihre weitere Behandlung’). Additionally, we decided to use one version for both inpatient and outpatient settings. We also rephrased the framing sentence according to suggestions of the participants. Questions 1 was well understood by all participants, so we did not have to change it. The different versions of question 2 were well understood by all participants. We choose the version, which was preferred by most participants and slightly changed wording according to participants’ suggestions. The versions of question 3 differed in their content but were also well understood by most participants. However, there were different opinions about the most relevant and useful version of question 3. We decided for the version of question 3, which was preferred by most of the participants. For the final version of the German Ask3Q, see Data S4.

### 3.2 Phase 2: Assessment of acceptability and feasibility

#### 3.2.1 Sample characteristics

Three focus groups with n = 24 patients with a cancer disease, one focus group with n = 5 physicians, and two focus groups with n = 13 nurses, as well as interviews with n = 1 physician and n = 2 nurses were conducted. Focus groups lasted 94 minutes on average. Interviews lasted 48 minutes on average. For sample characteristics, see Tables 1 and 2.

**Table 2:** Demographic data of participating healthcare professionals (HCPs) of focus groups and individual interviews with n = 15 nurses and n = 6 physicians

	Phase 2: HCPs of focus groups and interviews (n = 21)	
	Physicians (n = 6)	Nurses (n = 15)
	n (%)	n (%)
Age		
<30 years	1 (16.7)	0 (0.0)
31‐40 years	0 (0.0)	3 (20.0)
41‐50 years	3 (50.0)	5 (33.3)
>50 years	2 (33.3)	7 (46.7)
Gender		
Female	4 (66.7)	13 (86.7)
Male	2 (33.3)	2 (13.3)
Profession		
Junior hospital‐based physician	2 (33.3)	/
Senior hospital‐based physician	2 (33.3)	/
Physician in outpatient practice	2 (33.3)	/
Work experience		
<5 years	1 (16.7)	0 (0.0)
11‐20 years	2 (33.3)	3 (20.0)
>20 years	3 (50.0)	12 (80.0)

### 3.3 Acceptability of Ask3Q

#### 3.3.1 Advantages for patients

All participants positively appraised the German Ask3Q. According to most participants, Ask3Q has the potential to motivate patients to ask more questions and to actively engage in decision‐making processes.

*‘But certainly, there are patients, who are still scared and to encourage them with these materials to think about that they have the possibility to ask questions, I like that’. Focus group physicians, P04*

Furthermore, Ask3Q could be used as a tool for patients to structure their questions and invites them to make notes before or after consultations.

#### 3.3.2 Advantages for HCPs

Participants noted that Ask3Q can also be used as a tool for HCPs to help them structure medical encounters without forgetting important information.

*‘Basically, it would be very positive, if the doctor would hand out such things, because he would be guided himself, so to say. These are common questions, which means that he needs to reflect again “did I address it’"or rather ‘did I give the patient the chance," so it is kind of a control for the doctor’. Focus group patients 3, P04*

#### 3.3.3 Advantages for the patient‐HCP‐interaction

Some participants mentioned that providing Ask3Q posters or postcards in a hospital or practice might indicate HCPs patient‐centredness.

*‘If it [Ask 3 Questions] comes from the physician […] then you can already say that the doctor takes care of his patients’. Focus group patients 2, P06*

It might help to find the best treatment option for the patient and fosters trust in the individual physician and in the healthcare institution.

*‘I believe that this also creates trust, if such a poster would be now hanging in the waiting room of a practice at our place, patients would immediately have the feeling: ”Oh, they want to ‐ they promote my own self‐sufficiency ‐ my own self‐sufficient question‐asking behavior.” And I like that’. Focus group nurses 2, P03*

#### 3.3.4 Versatile usability

Most participants positively appraised that Ask3Q is a generic tool. They supported the idea that patients with different diseases in different stages as well as patients’ caregivers can use Ask3Q.

*‘And I also like that it is kept more general, that you don't need it just for one thing, or ‐ that you theoretically could take this card with you to any physicians consultation’. Focus group patients 1, P10*

#### 3.3.5 Wording and phrases

Participants differ in their opinion about single words and phrases. Some had the impression that important questions like ‘What is my disease?’ are missing.

*‘[This postcard] is a starting point, when a decision has to be made. But maybe a second postcard, with questions, "What can I do to positively influence the chemo therapy’?". Focus group patients 3, P04*

*‘I already have the first question. The diagnosis, ”What is my disease?”, the medical information, and then actually the second [question] will follow, “What are my options?”. That would be the order’. Focus group patients 3, P06*

Some participants claimed that patients might be irritated by the phrase ‘three important questions’ because they might have other questions more important to them or might have more than three questions.

*‘What bothers me about this card is the word “important”. […] So, when I see such a card, or see a poster, I immediately ask myself: "Are my questions actually important?’"And I immediately hold off myself’. Focus group patients 1, P01*

*‘Okay. It could of course be understood as “I am only allowed to ask three questions”’. Interview nurses 1*

Most participants highly valued the phrase ‘watch and wait’ because this option is often not discussed in clinical encounters. However, single participants argued that ‘watch and wait’ is a treatment option that physicians should mention by themselves, without the need to have this option spelt out in the question list.

*‘What I like very much is […] the phrase “inclusive watch and wait,” I think this is a very, very important supplement’. Focus group patients 3, P05*

*‘This is tendentious and that is not right. Well, that is not right and I also don't believe that physicians would like, if it is written like that, because that is already the answer […]’. Focus group nurses 2, P03*

Most participants and especially patients positively appraised question 3. However, some patients critically appraised that question 3 might be difficult to answer for physicians.

*‘But when I ask the physician: “How do I get support to help me make a decision that is right for me?”, the physician would say: “Yes, I, as your doctor, am the support”. That’s why you are here’. Focus group patients 2, P07*

Others make suggestions to improve specific phrases or questions.

*‘Every patient has to know that he is the one who has to decide what is happening to him. A physician just makes a suggestion. But you don´t have to follow. So, you might say: “Ask three important questions. You are the manager of your own health”’. Focus group patients 2, P03*

#### 3.3.6 Design

Participants differed in their opinion about the design of the Ask3Q intervention. Many participants liked the design of the posters and postcards, others had troubles with interpretation of the figures.

*‘I think this thing [the postcard/poster] is working well because it attracts attention with the picture’. Focus group patients 1, P10*

*‘When I am […] looking for help, I don´t want to do interpretations. Then, I want to get help’. Focus group patients 1, P03*

Several further suggestions (eg using different colours and bigger letters to increase readability) were made.

For the final coding scheme and additional quotes for the subcodes above, see Table 3.

**Table 3:** Final coding scheme for focus groups and interviews

A: Codes for the main section “Acceptability of Ask 3 Questions”		
A	Acceptability	
A1	Positive evaluation of Ask 3 Questions	‘So, I think this one ‐ I think this is great. So, someone who never had such a diagnosis, exactly needs these three things. I think this is very good’. *Focus group patients 1, P07*
A2	Function of Ask 3 Questions	
A2.1	Advantages for patients	
A2.1.1	Encourages patients to actively participate in decision‐making/ actively ask questions	‘These questions promote self‐confidence of the patient’. *Focus group nurses 2, P05* ‘But certainly, there are patients, who are still scared and to encourage them with these materials to think about that they have the possibility to ask questions, I like that’. *Focus group physicians, P04*
A2.1.2	Guideline for patients in a consultation	‘So I could imagine very well that the patient gets handed out this, thinks […] about it and can, during the doctor's consultation, during the discharge consultation or even at the beginning of the treatment, simply put it together again and then concretely work it off and read his questions and so on’. *Focus group patients 1, P02*
A2.1.3	For preparation and post processing of the consultation by the patient	‘I really like that, especially on the backside with the possibility to take notes. Even if it is not necessarily the answer to these questions, but maybe to some own questions. I think this could animate patients’. *Focus group nurses 1, P07*
A2.2	Advantages for healthcare professionals	
A2.2.1	Guideline for the physician in a consultation	‘Basically, it would be very positive, if the doctor would hand out such things, because he would be guided himself, so to say. These are common questions, which means that he needs to reflect again “did I address it” or rather “did I give the patient the chance,” so it is kind of a control for the doctor’. *Focus group patients 3, P04*
A2.3	Advantage for the patient‐healthcare professional interaction	
A2.3.1	Indicates patient‐centredness	‘If it [Ask 3 Questions] comes from the physician […] then you can already say that the doctor takes care of his patients’. *Focus group patients 2, P06*
A2.3.2	Helps to find the best treatment options	‘And that is what I had in mind when I read the invitation that this physician‐patient patient‐physician consultation would finally lead to better well‐being for the patient. That he will not be treated according to the method which is cheapest, tawdriest, fastest or something, but that the optimal method will be found. And of course this could only be found if I use what is written there [on the postcard]’. *Focus group patients 1, P02*
A2.3.3	Fosters trust into the physician/ into the practice	‘I believe that this also creates trust, if such a poster would be now hanging in the waiting room of a practice at our place, patients would immediately have the feeling: ‘Oh, they want to ‐ they promote my own self‐sufficient ‐ my own self‐sufficient question‐asking behavior.” And I like that’. *Focus group nurses 2, P03*
A3	Characteristics of Ask 3 Questions: Versatile usability	
A3.1	Can be used for different diseases/ in different stages of disease	‘And I also like that it is kept more general, that you don't need it just for one thing, or ‐ that you theoretically could take this card with you to any physicians consultation’. *Focus group patients 1, P10*
A3.2	Could also be used by relatives	‘And then also for the relatives, when they [the patients] are laying intubated on the intensive care unit, when everything went wrong, then it is just as important for the relatives’. *Focus group nurses 2, P04*
A4	Characteristics of Ask 3 Questions: Wording and phrases	
A4.1	Statements regarding the phrases in general	‘I think the questions are nicely phrased. Also not too long, so that everyone can actually read them when passing by, because you don't always have that much time as a patient […]’. *Interview physician*
A4.2	Postcard is not detailed enough; important content is missing	‘I would like to have more information here and I know, for example with an "e.g." and three dots at the end or so, that you just initiate things again’. *Focus group patients 3, P05* ‘[This postcard] is a starting point, when a decision has to be made. But maybe a second postcard, with questions, what can I do to positively influence the chemo therapy’. *Focus group patients 3, P04* ‘I already have the first question. The diagnosis, “What is my disease?”, the medical information, and then actually the second [question] will follow, “What are my options?”. That would be the order’. *Focus group patients 3, P06*
A4.3	Statements regarding specific phrases	
A4.3.1	Statements regarding the word ‘important’ (in the Ask 3 Questions title)	‘What bothers me about this card is the word "important". I think it is stupid. So, when I see such a card, or see a poster, I immediately ask myself: “Are my questions actually important?” And I immediately hold off myself’. *Focus group patients 1, P01*
A4.3.2	Statements regarding "three questions“ (in the Ask 3 Questions title)	‘Okay. It could of course be understood as “I am only allowed to ask three questions”’. *Interview nurses 1*
A4.3.3	Statements regarding „watch and wait“ (question 1)	‘What I like very much is […] the phrase ‘inclusive watch and wait’, I think this is a very, very important supplement’. *Focus group patients 3, P05* ‘This is tendentious and that is not right. Well, that is not right and I also don't believe that physicians would like, if it is written like that, because that is already the answer ‐ so yes. It feels like that’. *Focus group nurses 2, P03*
A4.3.4	Statements about question 3	‘The young woman, who is a single mother with three children at home and cannot concentrate on her disease at all, because she doesn't know how to manage it, right? So there ‐ the third point, I think, is actually always meaningful’. *Interview physician* ‘But when I ask the physician: “How do I get support to help me make a decision that is right for me?”, the physician would say: “Yes, I, as your doctor, am the support”. That’s why you are here.’ *Focus group patients 2, P07*
A4.4	Suggestions to improve specific phrases	‘Why, I ask myself, why is there not written “Where do I get support?”’ *Focus group patients 3, P02* ‘Every patient has to know that he is the one who has to decide what is happening to him. A physician just makes a suggestion. But you don´t have to follow. So, you might say: “Ask three important questions. You are the manager of your own health”’. *Focus group patients 2, P03*
A4	Characteristics of Ask 3 Questions: Design	
A4.1	Negative comments	‘When I am […] looking for help, I don´t want to do interpretations. Then, I want to get help’. *Focus group patients 1, P03*
A4.2	Positive comments	‘Also, nice this design, so the symbols on the top, symbolism. I like that’. *Focus group nurses 2, P06* ‘I think this thing [the postcard/poster] is working well because it attracts attention with the picture’. *Focus group patients 1, P10*
A4.3	Suggestions for improvement	‘Something I could give you for the layout or as a suggestion, would be the questions and below a larger space for writing, maybe also, that older people have more space, not always turn around [the postcard], drift away from the topic, so there is more structure, to have blank lines directly under the questions’. *Focus group patients 1, P02*

B: Codes for the main section ‘Feasibility of Ask 3 Questions’		
F	Feasibility	
F1	Facilitators for effective use of Ask 3 Questions tool	
F1.1	Characteristics of Ask 3 Questions	
F1.1.1	Materials need to attract attention	‘If I go to the pharmacy as a patient and see this postcard, if it does not make “Wow” for me and tells me, this can be something for me, then I will not take the postcard with me’. *Focus group patients 2, P03*
F1.1.2	Materials need to reach the target group specifically	‘And certainly, the card could be a little bit helpful, if you have had time to deal with it, but if it is just lying somewhere for the first consultation or so, I don't think a patient will notice it. Because the thoughts are somewhere else’. *Focus group patients 3, P07*
F1.2	Structural Requirements	
F1.2.1	Consultations must take place in an appropriate room	‘The best would be, if there is space for it, a room with four walls, where the neighbor does not hear everything and just a room, which – even a small time slot, where people can ask questions’. *Focus group nurses 2, P06*
F1.2.2	Time and staff for consultations must be available	‘So, one should have answers to the questions, than they should take some time, maybe one [patient] is really fast, and wants to finish soon, but the other one needs much information’. *Interview nurses 1*
F1.2.3	Patients need time for contemplation/ processing	‘So that, I think you can totally shorten it, people need time for contemplation, when they get the diagnosis, in fact it is not like that, they come, introduce themselves, “Tomorrow you will lay on the table [for surgery],” that´s it’. *Focus group nurses 1, P05*
F1.2.4	Incentives for physicians would be helpful	‘They [physicians] need to get money, then they will do it’. *Focus group patients 1, P01*
F1.3	Requirements regarding patients	
F1.3.1	Patients need access to information	‘And that's exactly how I think about this card, because if it is introduced properly and explained, then he will have a chance to use it’. *Focus group nurses 2, P01*
F1.3.2	Patients must be receptive/ not overwhelmed	‘You have to get that in this situation, all the excitement and nervousness is coming and then the patient ignores that [the postcard] and only talks about what is really important and other information passes by’. *Focus group nurses 1, P02*
F1.3.3	Patients must be self‐confident	‘Well, it is like, that the postcard is perfect for the so called “self‐confident” patient. But he already has to be self‐confident. Actually, he has to say “Wait a moment, I don't agree”’. *Focus group nurses 1, P04*
F1.3.4	Patients must be able and should want to participate in decision‐making	‘So, I am a little bit in doubt, whether you can really expect this from many people, to stand the many different existing options. That makes the decision even harder and I experience many patients, who also a – want someone else to show them a way’. *Focus group physicians, P03*
F1.4	Requirements regarding healthcare professionals	
F1.4.1	Healthcare professionals are willing to take a patient‐centred approach	‘It is not always at eye level, the conversation […] and sometimes hierarchy is also a really important part for some people, so’. *Interview nurses 2*
F1.4.2	Healthcare professionals are motivated to answer questions/ have the necessary knowledge to answer questions	‘Because they always said: “You [the patient] have to ask questions, you have to ask questions.” But at the same time, you have to say: “You [the physician] have to give answers, you have to give answers.” Physicians are taken into responsibility too little’. *Focus group patients 1, P10*
F1.4.3	Healthcare professionals are willing to use the Ask 3 Questions tool	‘It [the use of Ask 3 Questions] may be more realistic in one department, because people there are more open minded, also the physicians. But it might be more problematic in other departments and that is a bit my – in my opinion it may be difficult in some departments’. *Focus Group nurses 2, P04*
F1.5	Personal dissemination via physicians/ nurses	“When it comes directly from the doctor or in the conversation, it is something different. Then I will sit down, then I will probably also think about it. This definitely addresses me more than just a flyer displayed somewhere.” *Focus group patients 2, P02* One nurse made a suggestion how to introduce the postcard to patients: ‘We want to give you this short guideline. Whenever you have to make a decision concerning your treatment here in the hospital, you can use this for orientation’. *Focus group nurses 2, P04*
F1.5.1	Physicians have to give answers, nurses feel themselves overstrained when answering the questions	‘But the – the first medical contact person, normally this is the nurse – I would think and then you can make sure, that for example the doctor says again: “Did you get this card or have ‐”. It would also be useful, that the nurse says: “Read this and think about it.”, so that you can use the time’. *Interview, physician*
F2	Ways of dissemination of Ask 3 Questions	
F2.1	Dissemination via hospitals	
F2.1.1	Dissemination in in‐ward clinics	‘This should be displayed everywhere in the health care system, in every outpatient practice. Than, it will be recognized, patients will always be remembered’. *Focus group physicians, P02* ‘It belongs to the patient’s room, even more important than the “IGEL‐services,” which is offered there’. *Focus group patients 1, P02*
F2.1.1.1	Via the patient terminal next to the bed	‘There [via the patient terminal] are even more information, somehow. Among other things like I said, these surveys and I think other things as well, it is necessary to really get more into this, and then by this way it could be possible, [to inform someone] who never saw a waiting room’. *Interview nurses 2*
F2.1.1.2	As part of informed consent sheets and anamnesis sheets or in the admission folder	‘I thought, well actually ‐ actually one could even imagine to integrate these three questions into informed consent and anamnesis sheets, right? So, for ‐ for both [physicians and patients] as a support and that you have to kind of tick it off’. *Focus group physicians, P02*
F2.1.2	Dissemination in outpatient practices/ ambulances	‘So, I also believe that the postcard actually makes a much greater difference and has a greater effect in practices and ambulances, than in our in‐ward clinic’. *Focus group nurses 2, P01* ‘That's why I said, in the polyclinic, because there is still time, so that means, now it is not life‐threatening, then the patients also go home with the, possibly already with an admission appointment, but that can also be cancelled, even that happens’. *Focus group nurses 1, P03*
F2.1.2.1	Presentation in waiting rooms	‘And of course, the idea of present something like this in the waiting room is good’. *Focus group patients 1, P02*
F2.1.3	As enclosure in letters to patients	‘Well, you could also do it in the written contact’. *Interview nurses 1*
F2.1.4	Via Internet/ Intranet of the hospital	‘And maybe post this in the intranet, where a physician can also say, okay, I will print it out and hand it out to the patients’. *Focus group nurses 1, P02*
F2.2	Dissemination via self‐help groups	‘One could also use this in the self‐help groups. Especially there, so that you encourage them again, to say: "Come on, it is like that now, you are allowed to ask questions." So that they can practice that even more. And to encourage them again’. *Focus group patients 1, P03*
F2.3	Dissemination via health insurances	‘Actually, such a thing could be disseminated by the health insurgencies, to all their members. That ‐ so you regularly get a new insurance card and so on. And then one could actually – this is technically true for every disease’. *Focus group patients 1, P08*
F2.4	Dissemination via journals/ magazines	‘Under circumstances there may be an advertisement in a journal. In magazines, if it is a poster, which is plausible, when it is finished later’. *Focus group patients 2, P04*
F2.5	Dissemination via screening‐centres	‘Well, I thought about those screening‐centers as another possible place for example’. *Focus group patients 2, P07*
F2.6	Dissemination via pharmacies	‘There [in hospitals and practices] is a lot of information material presented and it would be very possible that such a postcard could get lost. That is why pharmacies maybe would be good’. *Focus group patients 2, P03*
F2.7	Dissemination via a big public campaign	‘I think there is need of a huge monster‐campaign. Seriously, before "Tatort" [a popular German crime series on TV], after "Tatort" and before "Deutschland sucht den Superstar" [a popular German casting show on TV]’. *Focus group patients 1, P01*
F2.8	Dissemination via pharmaceutical companies	‘Secondly, it usually always works with a pharma[ceutical company]. They usually catch the physicians’. *Focus groups patients 1, P04*
F2.9	Printing and Dissemination via bookmarks	‘Yes, it is an older medium, I would say, but I just thought about ‐ as bookmark in books ‐ bookshops or so’. *Focus group patients 2, P02*
F3	Time points for dissemination of Ask 3 Questions	
F3.1	Before diagnosis	‘I think we are too late for this. We have to pick up the patients earlier, the person who realizes there is something wrong with him, this is the person I mean, which we have to pick up earlier’. *Focus group patients 2., P05*
F3.2	At diagnosis	‘But still it is my opinion, it is mainly for a first consultation ‐ that it is important, because sooner or later, when you have more appointments, you already informed yourself a bit and know for yourself and can also explicitly obtain the answers to other important questions’. *Focus group patients 1, P10*
F3.3	At admission to inpatient care	‘In the in‐ward context, perhaps we rather have the advantage, that we can hand it out during the admission process, we work a lot with brochures, with information material, which should encourage the patient to participate with the whole diagnosis and therapy process actively’. *Focus group nurses 1, P07*
F3.4	During in‐ward stay	‘So, I also think it would be quite good to simply hand them [the postcards] out during a medical round and then the patients can think about’. *Focus group nurses 1, P02*
F3.5	At discharge from the hospital	‘So I could imagine very well that the patient gets handed out this, thinks […] about it and can, during the doctor's consultation, during the discharge consultation or even at the beginning of the treatment, simply put it together again and then concretely work it off and read his questions and so on’. *Focus group nurses 1, P02*
F3.6	Whenever a decision has to be made	‘As a cancer patient, there is a new situation every month, where I have to adjust and prior to death nearly every day or so. […] They are basically brought into action by these key questions, into reflection – How will it go on?’ *Focus group nurses 2, P05*
F3.7	Situation of disease	
F3.7.1	There should be several treatment options	‘The [treatment] options and the pros and cons of course particularly make sense, if there are true alternatives’. *Interview physicians*
F3.7.2	Patients should not be too ill/ no emergency situation	‘If someone is really ill now, I think, then he won´t really think about the three questions’. *Focus group nurses 1, P05*

**Figure 1:**
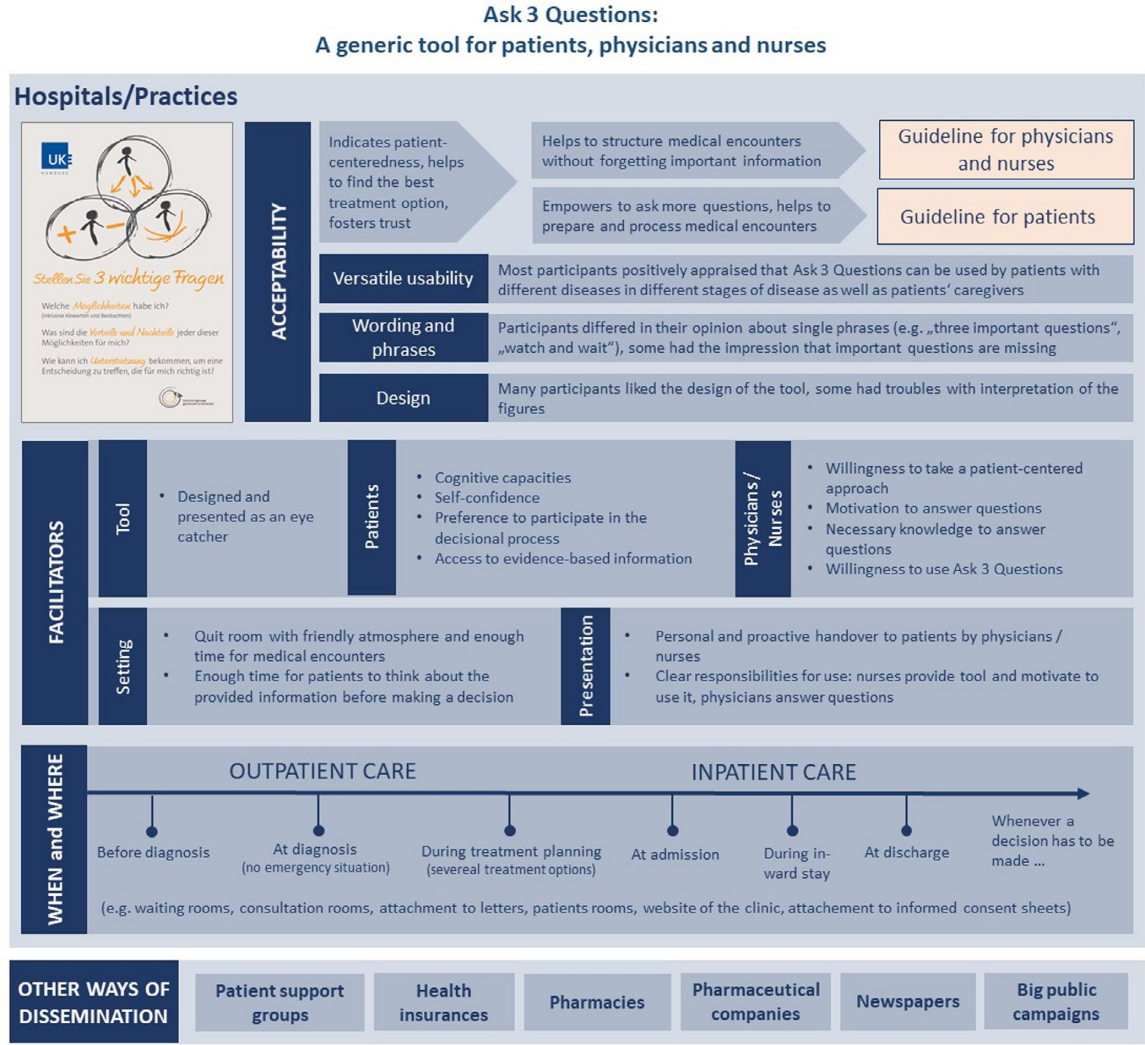
Summarized results of qualitative evaluation of acceptability and feasibility of Ask 3 Questions

### 3.4 Feasibility of Ask3Q

#### 3.4.1 Facilitators for effective use of Ask3Q

Characteristics of Ask3Q, the setting, patients’ characteristics, HCPs’ attitudes and behaviour and personal dissimination might facilitate the use of Ask3Q.

First, Ask3Q posters and postcards have to be designed and presented as an eye‐catcher to directly attract the attention of the target group.

*‘If I go to the pharmacy as a patient and see this postcard, if it does not make “Wow” for me and tells me, this can be something for me, then I will not take the postcard with me’. Focus group patients 2, P03*

Second, the setting and the available amount of time have to be appropriate. Especially conversations about treatment options and healthcare decisions should take place in a quiet room with friendly atmosphere.

*‘The best would be, if there is space for it, a room with four walls, where the neighbor does not hear everything and just a room, which – even a small time slot, where people can ask questions’. Focus group nurses 2, P06*

Afterwards, patients should be offered enough time to think about the provided information before making a decision.

*‘So that, I think you can totally shorten it, people need time for contemplation, when they get the diagnosis, in fact it is not like that, they [the doctors] come, introduce themselves, “Tomorrow you will lay on the table [for surgery],” that´s it’. Focus group nurses 1, P05*

Third, characteristics of patients like cognitive capacities, self‐confidence and a preference to participate in the decisional process might facilitate the use of Ask3Q.

*‘Well, it is like, that the postcard is perfect for the so called “self‐confident” patient. But he already has to be self‐confident. Actually, he has to say “Wait a moment, I don't agree”’. Focus group nurses 1, P04*

*‘So, I am a little bit in doubt, whether you can really expect this from many people, to stand the many different existing options. That makes the decision even harder and I experience many patients, who also a – want someone else to show them a way’. Focus group physicians, P03*

Fourth, HCPs should be willing to take a patient‐centred approach, be motivated to answer questions, have the knowledge to answer questions and should be willing to use Ask3Q.

*‘Because they always said: “You [the patient] have to ask questions, you have to ask questions.” But at the same time you have to say: “You [the physician] have to give answers, you have to give answers.” Physicians are taken into responsibility too little’. Focus group patients 1, P10*

*‘It [the use of Ask 3 Questions] may be more realistic in one department, because people there are more open minded, also the physicians. But it might be more problematic in other departments and that is a bit my – in my opinion it may be difficult in some departments’. Focus group nurses 2, P04*

Fifth, the use of Ask3Q can be facilitated if HCPs hand over the postcards personally and proactively to their patients. It would show patients that asking questions and being actively involved in the patient‐physician interaction is important and desired by the institution.

*‘When it comes directly from the doctor or in the conversation, it is something different. Then I will sit down, then I will probably also think about it. This definitely addresses me more than just a flyer displayed somewhere’. Focus group patients 2, P02*

One nurse made a suggestion how to introduce the postcard to patients:

*‘We want to give you this short guideline. Whenever you have to make a decision concerning your treatment here in the hospital, you can use this for orientation’. Focus group nurses 2, P04*

Nurses and physicians agreed that it is important to have clear responsibilities for dissemination of the postcards to patients. Nurses primarily see their role in providing information material, handing over the postcards and motivating to ask questions in the patient‐physician interaction. Most nurses and physicians see it as task of the physicians to answer the three questions.

*‘But the – the first medical contact person, normally this is the nurse – I would think and then you can make sure, that for example the doctor says again: “Did you get this card or have –“. It would also be useful, that the nurse says: "Read this and think about it.", so that you can use the time’. Interview, physician*

#### 3.4.2 Ways of dissemination of Ask3Q

While participants came up with a range of ideas to disseminate Ask3Q (eg patient support groups, health insurances, newspapers, pharmacies, pharmaceutical firms, big public campaigns), the focus of the discussions targeted dissemination of Ask3Q in hospitals and outpatient practices. Ask3Q can be presented to inpatients (eg in patients’ rooms, as an attachment to information material or informed consent sheets) and outpatients (eg in waiting rooms, consultation rooms), via letters to patients, and on the website of the practice or hospital.

*‘This should be displayed everywhere in the health care system, in every outpatient practice. Then, it will be recognized, patients will always be remembered’. Focus group physicians, P02*

Most participants prefer provision of Ask3Q during outpatient consultations, where patients are most often confronted with their diagnosis, treatment options and important decisions regarding their healthcare.

*‘That's why I said, in the polyclinic, because there is still time, so that means, now it is not life‐threatening, then the patients also go home with the ‐ possibly already with an admission appointment, but that can also be cancelled, even that happens’. Focus group nurses 1, P03*

#### 3.4.3 Time points for dissemination of Ask3Q

Some participants suggested that Ask3Q could be helpful at any time prior to diagnosis, during the first consultation, during the admission process to inpatient care, during ward rounds and at discharge from the hospital. Many participants supported the idea that Ask3Q should be provided as soon as possible and whenever a decision has to be made.

*‘As a cancer patient, there is a new situation every month, where I have to adjust and prior to death nearly every day or so. […] They are basically brought into action by these key questions, into reflection – How will it go on?’ Focus group nurses 2, P05*

In emergency situations or in case of lack of treatment alternatives, the use of Ask3Q was perceived as inapplicable.

*‘If someone is really ill now, I think, then he won´t really think about the three questions’. Focus group nurses 1, P05*

For the final coding scheme and additional quotes for the subcodes above, see Table 3.

Figure 1 summarizes the results of the qualitative evaluation of acceptability and feasibility of Ask3Q.

## 4 DISCUSSION

Within this qualitative study, we provided the first German version of Ask3Q. We reached consensus for translation of the available English Ask3Q versions within the first round of discussion. Via cognitive interviews, we constructed a final German Ask3Q version, which was well understood by patients with a cancer disease and considered most useful. In focus groups and interviews, participants agreed that Ask3Q has the potential to empower patients by motivating them to ask more questions in medical encounters and might remind HCPs to provide important information. Participants agreed that implementation of Ask3Q in the German healthcare system is feasible. Our study identified several facilitators for successful implementation of Ask3Q, including characteristics of patients, the setting, and HCPs’ attitude and behaviour. If Ask3Q is endorsed by HCPs and handed to patients proactively and personally, this might facilitate use of Ask3Q by patients. Clear responsibilities for dissemination of Ask3Q might increase feasibility and effectiveness of Ask3Q. For implementation of Ask3Q, the appropriate time and ways of dissemination have to be considered. Patients should receive Ask3Q as soon as possible, ideally before getting a diagnosis and whenever a decision has to be made. While Ask3Q is designed as a generic instrument,^23^ which can be used for different diseases and at different time points prior, during, and after a diagnosis, it is probably of highest relevance when a health‐related decision has to be made. Our results revealed that over the course of cancer disease trajectory, there are a range of other questions apart from decision‐making that patients want to ask. However, short QPLs like Ask3Q have shown to improve question‐asking behaviour and patient participation in decision‐making in general, even if the specific questions of the QPL have not been asked by patients.^18^, ^20^, ^25^, ^53^

Encouraging patients to be more active in clinical consultations and to ask a few key questions can support SDM adoption of HCPs.^11^, ^25^ Our findings suggest that the German Ask3Q has the potential to decrease the asymmetry of patient‐physician communication. Thereby, it could foster SDM in clinical consultations and in the interaction between nurses and patients. But interventions to improve communication are most likely to be effective if they address both patients and HCPs and are actively promoted and handed over by HCPs.^54^, ^55^ It might even have negative effects if interventions focus only on patients.^56^, ^57^ Thus, it is an important advantage of the German Ask3Q that it addresses not only patients, but can also be used by HCPs.

It is likely that successful implementation of Ask3Q might come along with changes in clinical practice and is determined by organizational culture.^58^ High acceptability of the intervention, as found in this study, is an important implementation facilitator.^59^ But when implementing Ask3Q in a clinical setting, it has to fit into the current clinical system, should have minimal impact on work practices, should be promoted by clinical champions, and developed and disseminated by the medical staff.^18^, ^59^, ^60^

Besides the developed German version of Ask3Q, the thorough translation, adaptation and evaluation process can be used to inform subsequent translations and cross‐cultural adaptations of Ask3Q as well as other similar interventions. Additionally, the results of our qualitative evaluation can be used to plan following implementation studies in German‐speaking countries as well as worldwide. Additionally, our final coding scheme can be used to define deductive categories for qualitative content analysis in further studies evaluating Ask3Q.

Yet, we need to study whether effects of Ask3Q on patient participation in decision‐making, question‐asking behaviour of patients and communication behaviour of HCPs can be replicated in Germany.^25^, ^26^, ^27^, ^29^ Furthermore, there might be other barriers or facilitators for successful implementation of Ask3Q, which are difficult to anticipate. This has to be evaluated in future implementation studies.

### 4.1 Strengths and limitations

A strength of this study is its qualitative approach. In planning, conducting and reporting this study, we considered credibility, transferability, neutrality and dependability of our findings. Thereby, we could generate insights into acceptability and feasibility of Ask3Q from the target populations’ view. To increase robustness of results, data analysis was conducted by two study team members and the refined coding scheme was re‐applied to all the data.

This study comes along with several limitations. First, we did not perform a third round of cognitive interviews to test the final adaptations of Ask3Q. Second, we used a convenience sampling approach which might come along with a self‐selection bias of participants interested in the topic. Additionally, most participants were patients and HCPs at one Comprehensive Cancer Center in Hamburg, Germany. Therefore, our findings might not be generalizable to other healthcare systems and/or other countries. Third, most patients were highly educated and native German speakers. Further research should evaluate the intervention in a more diverse population, including vulnerable populations.^61^ Additionally, we found a mismatch between the educational level and health literacy of patients taking part in focus groups. 62.5% of patients were highly educated but only 29.2% showed adequate health literacy. Despite the HLS‐EU‐Q16 is a well‐established measure,^62^, ^63^, ^64^, ^65^ it was recently criticized for being not valid according to the concept of evidence‐based medicine.^66^, ^67^ Since it was beyond the scope of this study, this should be analysed and discussed in further validation studies of the HLS‐EU‐Q16. Fourth, it was difficult to recruit physicians for focus groups, leading to an overrepresentation of nurses in the sample of HCPs. Lack of time, less acceptability of the Ask3Q intervention, less interest in the topic or a too small financial incentive might be reasons for the low participation rate of physicians.^68^ Nevertheless, since a broad range of sensitive topics were addressed in the group discussions, problems in the recruitment process might not have influenced the depth and quality of our data.^69^

## 5 CONCLUSION

We provide the German version of Ask3Q. The German Ask3Q has the potential to empower patients and guide HCPs throughout medical encounters. Our study participants agreed that implementation of Ask3Q in the German healthcare system is feasible. Several facilitators like clear responsibilities have to be considered. Future studies should evaluate if positive effects of Ask3Q can be replicated for patient participation in decision‐making of patients and communication behaviour of HCPs in Germany.

## CONFLICT OF INTERESTS

PH gave one scientific presentation on shared decision‐making during a lunch symposium in 2018, for which she received compensation and travel compensation from GlaxoSmithKline GmbH. AL, WF, CT, MF and IS declared to have no competing interests.

## AUTHORS’ CONTRIBUTIONS

AL, PH and IS made substantial contributions to the design and preparation of the study. AL, PH, WF and IS collected the data. AL conducted the analysis in collaboration with CT and MR. All authors contributed to the interpretation of results. AL drafted the manuscript and PH, IS, WF, CT and MR were involved in critically revising the manuscript for important intellectual content. All authors gave final approval of the version to be published.

## ETHICS APPROVAL AND CONSENT TO PARTICIPATE

The study was approved by the Ethics Committee of the Medical Association Hamburg (Germany, study ID PV5368). The study was carried out in accordance to the latest version of the Helsinki Declaration of the World Medical Association. Principles of good clinical practice were respected. Data protection requirements were met. Study participation was voluntary. Participants received oral and written information and had the possibility to ask further questions. They were informed about the data protection police of the study and gave informed consent to the recordings of the focus groups and interviews and to the recordings to be transcribed verbatim.

## Supporting information

Supplementary File S1

Supplementary File S2

Supplementary File S3

Supplementary File S4

## Data Availability

The data set collected and analysed during this study is available from the corresponding author on reasonable request due to ethical and privacy restrictions.
