# Supplementary material for: Adaptation and qualitative evaluation of Ask 3 Questions — a simple and generic intervention to foster patient empowerment
Source: Health Expect. 2020 Aug 1;23(5):1310–25. doi: 10.1111/hex.13114 (PMC7696208; doi:10.1111/hex.13114)
Supplement: Supplementary file 2 — Supplementary File S2 [file HEX-23-1310-s002.docx]

**Supplementary File 2: Details on researcher characteristics, recruitment process, data collection and guideline of focus groups and interviews**

**A: Researcher characteristics**

AL is a female neurocognitive psychologist (Dipl., M.Sc.), doctoral researcher, and psycho-oncologist. She is experienced in interviewing, but was a first-time moderator of focus groups. She received training on moderating focus groups and qualitative content analysis prior to data collection and analysis. IS is a female clinical psychologist (Dipl.-Psych.) and senior researcher, trained psychotherapist, and psycho-oncologist. PH is a female clinical psychologist (Dipl.-Psych.), at the time of data collection and analysis a doctoral researcher, psychotherapist in training, and psycho-oncologist. WF is a female health scientist (M.Sc.) and doctoral researcher. IS, PH and WF have comprehensive experiences in moderating focus groups and conducting qualitative data analysis. LD and CT are undergraduate students in psychology. MR has a bachelor’s degree in health sciences and is a graduate student in health sciences. LD, CT and MR had no experiences in focus groups or qualitative content analysis prior to data collection.

**B: Recruitment process**

**Recruitment for cognitive interviews (phase 1).** 21 patients with a cancer disease were invited to take part in cognitive interviews. Eight patients were not interested in participation, three patients were interested but could not take part due to illness or reasons of time and refused participation before signing the informed consent sheet. Finally, ten patients took part in cognitive interviews and signed the informed consent sheet.

**Recruitment for focus groups / individual interviews (phase 2).** We followed a convenience sampling approach. Patients had to be 18 years or older and diagnosed with cancer prior to participation. We recruited patients via (1) the psycho-oncological outpatient clinic of the University Medical Center Hamburg-Eppendorf, (2) patient information events at the Hamburg Cancer Society and the University Medical Center Hamburg-Eppendorf, and (3) patient support groups for patients with a cancer disease in Hamburg. We invited physicians and nurses by sending an e-mail to head physicians and leading nurses of all clinics of the University Medical Center Hamburg-Eppendorf except of clinics, which take part in the actually running SDM implementation study, where the Ask 3 Questions intervention is part of the implementation program [1]. We also invited physicians and nurses of outpatient oncology practices in Hamburg, Germany, by sending an e-mail and an invitation letter to these outpatient oncology practices. E-mails were either forwarded to the employees of the clinics and/or the study team was invited to present the study personally e.g. during a team meeting. In case of no answer, reminders were send via e-mail and/or leading nurses were contacted via phone. Physicians and nurses, who were interested in participation but could not take part at the announced dates, were offered an individual interview. There were no relationships to participants established prior to study commencement.

**Non-participation in focus groups and interviews (phase 2).** Due to the recruitment process, it is not possible to evaluate how many patients, nurses and physicians received the study invitation and were eligible but did not want to participate. 35 patients with a cancer disease contacted the study team to take part in a focus group. Out of this group, eleven patients refused taking part in a focus group before signing the informed consent sheet. 24 patients took part in the focus groups and signed the informed consent sheet. 20 nurses contacted the study team to take part in the study. Out of this group five nurses finally refused taking part before signing the informed consent sheet. 15 nurses took part in the focus groups / interview and signed the informed consent sheet. Seven physicians contacted the study team to take part in the study. Out of this group, one physician refused taking part before signing the informed consent sheet. 6 physicians took part in the focus groups / interview and signed the informed consent sheet. Reasons of patients, nurses and physicians for not taking part in the study after contacting the study team were lack of time, illness or unknown reasons.

**C: Setting of data collection**

Data for cognitive interviews and focus groups were collected in a seminar room of the Department of Medical Psychology at the UKE, Hamburg, Germany. Data for individual interviews were collected in the offices of the interviewee. During cognitive interviews, only the participant and the researcher were present. In focus groups, there were two moderators (researcher of the study team) and one minute taker besides the participants. In two individual interviews, only the participant and the researcher were present. In one interview, additionally a student assistant was present.

No interviews or focus groups were repeated. Transcripts were not returned to participants for comments and / or correction. Participants were also not invited to provide feedback on the finding.

**D: Guideline for interviews and focus groups**

Table A: Guideline for interviews and focus groups with patients, nurses and physicians

| **1.** | **Giving background information about the study and the study team**   - Short description of background and aims of the study (these information were also given in the informed consent sheets) - Moderators introduced themselves (e.g. name, profession, role in the study team, years of working in the department) |
| --- | --- |
| **2.** | **Handing out Ask3Q postcards / poster** |
| **3.** | **Giving background information about Ask 3 Questions (by study team):**   - - - - Material was developed in Australia and used in many countries.       - Translated from English into German for the first time by the study team       - Poster and postcards are used in a current study and are presented in practices, outpatient clinics, and inpatient wards, postcards can be displayed or handed out to the patients directly.       - We would like to know, what patients and healthcare professionals think about this material. |
| **4.** | **Questions about the presented material *[acceptability of the intervention]:***   - - - - What is your first impression of these posters and postcards?       - *Patients*: Would you like to use these materials in a clinical consultation? *Physicians/nurses*: Would you like to use these materials in your daily work?       - When would this material be helpful to you? Which aspects are especially relevant to you?       - When would the material be less helpful? |
| **5.** | **Questions about the use of the material *[feasibility of the intervention]*:**   - - - - Do you have any ideas, how or where these posters and postcards could also be used?       - Which conditions must be met in hospitals and practices, to use the material for patient empowerment in a reasonable way? |
| **6.** | **Questions about patient activation in general:**   - - - - What do you think about the idea of encouraging patients to ask questions?   In your opinion, how can patients be motivated to ask questions?   - - - - *Patients*: What would encourage you, to make decisions together and on eye-level with healthcare professionals?   *Physicians/nurses*: What would encourage you, to make decisions together and on eye-level with patients? |
| **7.** | **Conclusion**   - - - - If you think about all the aspects we talked about today, which are especially important to you?       - Is there something that we overlooked or that we should have talked about? |
