# Supplementary material for: Adaptation and qualitative evaluation of Ask 3 Questions — a simple and generic intervention to foster patient empowerment
Source: Health Expect. 2020 Aug 1;23(5):1310–25. doi: 10.1111/hex.13114 (PMC7696208; doi:10.1111/hex.13114)

**Supplementary File 3: Design of poster and postcards of the Ask 3 Questions intervention**

Figure A: Poster “Stellen Sie 3 wichtige Fragen” (engl. „Ask 3 Questions“)


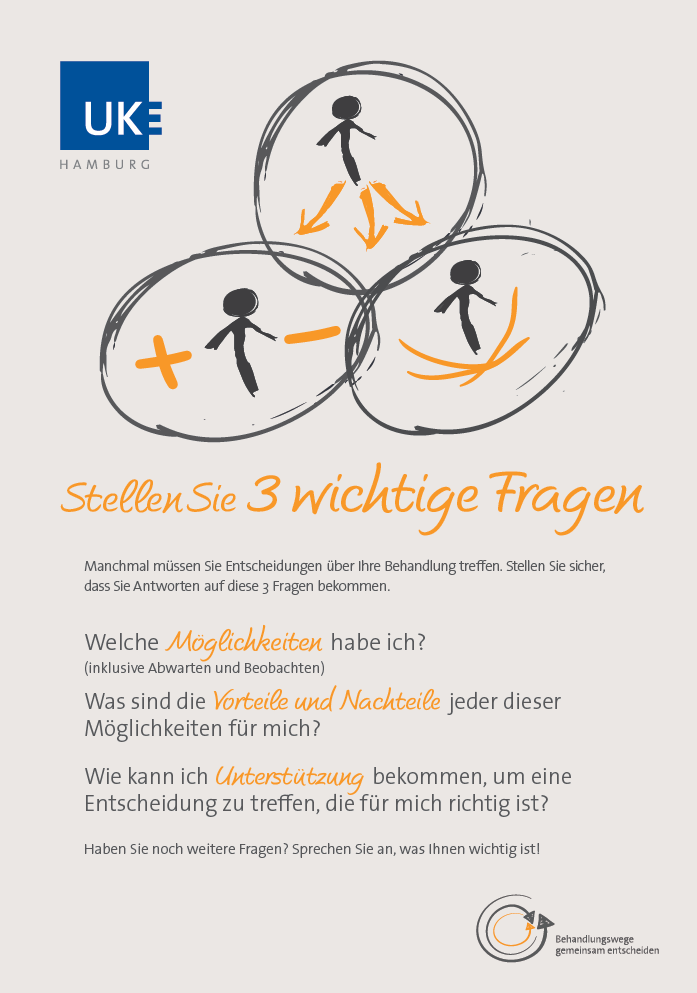


Figure B: Postcard (frontside and backside) “Stellen Sie 3 wichtige Fragen” (engl. „Ask 3 Questions“)


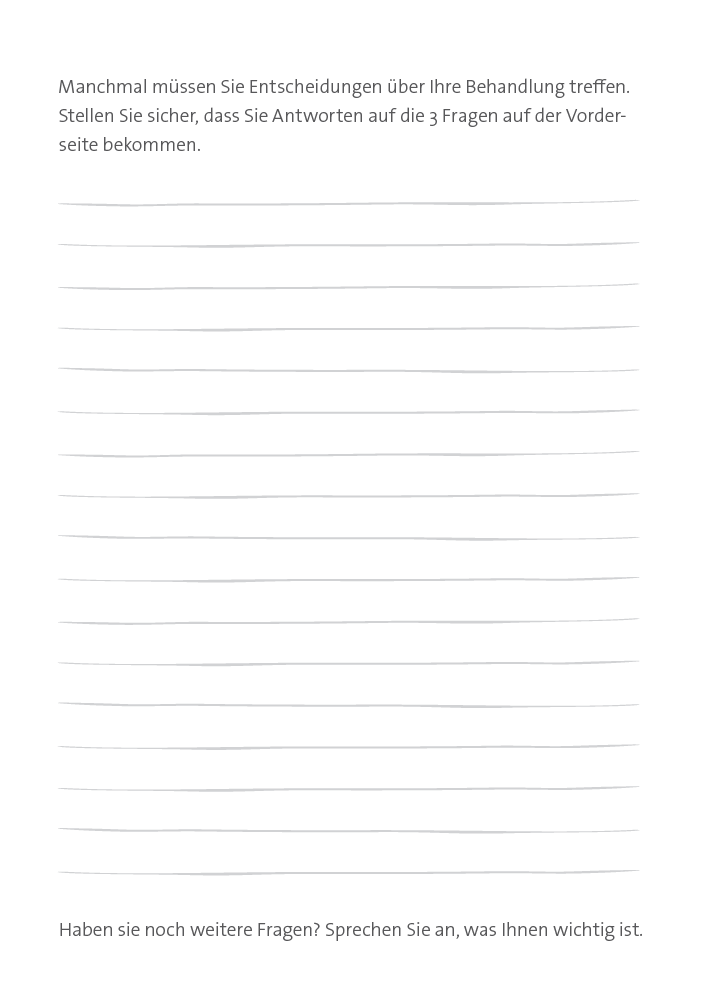

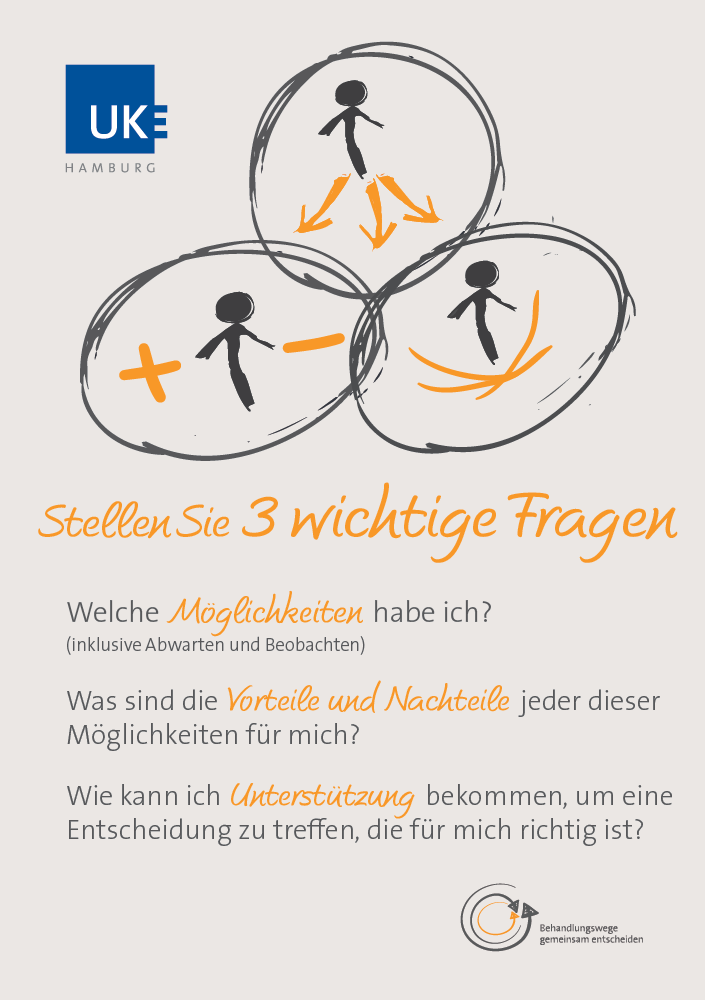

Supplement: Supplementary file 3 — Supplementary File S3 [file HEX-23-1310-s003.docx]
