# Supplementary material for: Adaptation and qualitative evaluation of Ask 3 Questions — a simple and generic intervention to foster patient empowerment
Source: Health Expect. 2020 Aug 1;23(5):1310–25. doi: 10.1111/hex.13114 (PMC7696208; doi:10.1111/hex.13114)
Supplement: Supplementary file 4 — Supplementary File S4 [file HEX-23-1310-s004.docx]

**Supplementary File 4: Details on translation process**

Table A: Currently available English versions of the Ask 3 Questions intervention and German translation.

|  | **References** | **Original version** | **Translated versions used for cognitive interviews** | **Final version after cognitive interviews** |
| --- | --- | --- | --- | --- |
| Title |  | Ask 3 Questions | Stellen Sie 3 Fragen | Stellen Sie 3 wichtige Fragen |
| Introduction | ^1^ ^2^ | Sometimes there will be choices to make about your healthcare. If you are asked to make a choice, make sure you get the answer to these 3 questions. | Manchmal müssen Sie während Ihres Termins [ambulant] / Krankenhausaufenthaltes [stationär] Entscheidungen über Ihre Gesundheitsversorgung treffen. Stellen Sie sicher, dass Sie Antworten auf diese 3 Fragen bekommen. | Manchmal müssen Sie Entscheidungen über Ihre weitere Behandlung treffen. Stellen Sie sicher, dass Sie Antworten auf diese 3 Fragen bekommen. |
|  | ^3^ | Normally there will be choices to make about your healthcare. Make sure you get answers to these three questions. |  |  |
|  | ^3^ | During your appointment, you might have to make choices about your healthcare. To help you make a decision and make the most of your appointment time, you may want to ask these three questions: |  |  |
|  | ^4^ | Three questions to ask your health professional. |  |  |
| Framing sentences | ^1^ ^2^ | We want to know what´s important to you. | Wir möchten wissen, was Ihnen wichtig ist. | Haben Sie noch weitere Fragen? Sprechen Sie an, was Ihnen wichtig ist! |
|  | ^3^ | Your health professional needs you to tell them what is important to you. |  |  |
| Question 1 | ^4^ | What are my options? (Including wait and watch) | Welche Möglichkeiten habe ich? (inklusive Abwarten und Beobachten) | Welche Möglichkeiten habe ich? (inklusive Abwarten und Beobachten) |
|  | ^2,3,5–7^ | What are my options? | Welche Möglichkeiten habe ich? |  |
| Question 2 | ^4,5^ | What are the possible benefits and harms of those options? | Was sind die möglichen Vorteile und Nachteile dieser Möglichkeiten? | Was sind die Vorteile und Nachteile jeder dieser Möglichkeiten für mich? |
|  | ^1,7^ | What are the possible benefits and risks of those options? |  |  |
|  | ^2^ | What are the possible benefits and risks? | Was sind die möglichen Vorteile und Nachteile? |  |
|  | ^3^ | What are the pros and cons of each option for me? | Was sind die möglichen Vorteile und Nachteile jeder dieser Möglichkeiten für mich? |  |
| Question 3 | ^4^ | How likely are each of those benefits and harms to happen to me? | Wie wahrscheinlich werden diese Vorteile und Nachteile bei mir auftreten? | Wie kann ich Unterstützung bekommen, um eine Entscheidung zu treffen, die für mich richtig ist? |
|  | ^5^ | How likely are each of those benefits and harms to happen to me? (Including: What will happen if I do nothing?) | Wie wahrscheinlich werden diese Vorteile und Nachteile bei mir auftreten? (inklusive: Was passiert, wenn ich nichts mache?) |  |
|  | ^7^ | How likely are the benefits and risks of each option to occur? | Wie wahrscheinlich ist es, dass diese Vorteile und Nachteile auftreten? |  |
|  | ^3^ | How do I get support to help me make a decision that is right for me? | Wie kann ich Unterstützung bekommen um eine Entscheidung zu treffen, die für mich richtig ist? |  |
|  | ^2,8^ | How can we make a decision together that is right for me? | Wie können wir gemeinsam eine Entscheidung treffen, die für mich richtig ist? |  |

**REFERENCES**

1. Lloyd A, Joseph-Williams N, Edwards A, Rix A, Elwyn G. Patchy “coherence”: Using normalization process theory to evaluate a multi-faceted shared decision making implementation program (MAGIC). Implement Sci. 2013;8:102.

2. King E, Taylor J, Williams R, Vanson T. The MAGIC Programme: Evaluation An Independent Evaluation of the MAGIC (Making Good Decisions in Collaboration) Improvement Programme. London: Health Foundation; 2013.

3. NHS. Ask 3 Questions. https://www.aquanw.nhs.uk/resources/shared-decision-making/Ask 3 Questions Poster.pdf. Accessed October 16, 2019.

4. CeMPED SU& FPN. ask share know. https://askshareknow.com.au/wp-content/uploads/2017/06/ASK_FLYER_green.pdf. Accessed October 16, 2019.

5. Shepherd HL, Barratt A, Jones A, et al. Can consumers learn to ask three questions to improve shared decision making? A feasibility study of the ASK (AskShareKnow) Patient-Clinician Communication Model intervention in a primary health-care setting. Heal Expect. 2016;19(5):1160-1168.

6. Shepherd HL, Barratt A, Trevena LJ, et al. Three questions that patients can ask to improve the quality of information physicians give about treatment options: A cross-over trial. Patient Educ Couns. 2011;84(3):379-385.

7. Case Study: Developing the ‘Ask 3 Questions’ Campaign to Raise People’s Awareness of Shared Decision Making. London: The Health Foundation; 2013.

8. Case Study: Testing and Refining the ‘Ask 3 Questions’ Campaign Promoting Shared Decision Making to Patients in Newcastle. London: The Health Foundation; 2013.
